# In Vivo Biocompatibility of *Synechococcus* sp. PCC 7002-Integrated Scaffolds for Skin Regeneration

**DOI:** 10.3390/jfb15100295

**Published:** 2024-10-03

**Authors:** Benedikt Fuchs, Sinan Mert, Constanze Kuhlmann, Alexandra Birt, Daniel Hofmann, Paul Severin Wiggenhauser, Riccardo E. Giunta, Myra N. Chavez, Jörg Nickelsen, Thilo Ludwig Schenck, Nicholas Moellhoff

**Affiliations:** 1 Division of Hand, Plastic and Aesthetic Surgery, LMU University Hospital, LMU Munich, 80336 Munich, Germany; sinan.mert@med.uni-muenchen.de (S.M.); constanze.kuhlmann@med.uni-muenchen.de (C.K.); alexandra.birt@med.uni-muenchen.de (A.B.); daniel.hofmann@med.uni-muenchen.de (D.H.); severin.wiggenhauser@med.uni-muenchen.de (P.S.W.); riccardo.giunta@med.uni-muenchen.de (R.E.G.); nichlas.moellhoff@med.uni-muenchen.de (N.M.); 2 Institute of Anatomy, University of Bern, 3012 Bern, Switzerland; myra.chavezrosas@unibe.ch; 3 Molecular Plant Science, Department Biology I, LMU Munich, 80336 Munich, Germany; joerg.nickelsen@lrz.uni-muenchen.de; 4 Praxis Dr.Schenck, Prinzregentenstraße 64, 80336 Munich, Germany; praxis@drschenck.com

**Keywords:** cyanobacteria, *Synechococcus* sp. PCC 7002, scaffolds, tissue engineering, biomaterials, biocompatibility

## Abstract

Cyanobacteria, commonly known as blue-green algae, are prevalent in freshwater systems and have gained interest for their potential in medical applications, particularly in skin regeneration. Among these, *Synechococcus* sp. strain PCC 7002 stands out because of its rapid proliferation and capacity to be genetically modified to produce growth factors. This study investigates the safety of *Synechococcus* sp. PCC 7002 when used in scaffolds for skin regeneration, focusing on systemic inflammatory responses in a murine model. We evaluated the following three groups: scaffolds colonized with genetically engineered bacteria producing hyaluronic acid, scaffolds with wild-type bacteria, and control scaffolds without bacteria. After seven days, we assessed systemic inflammation by measuring changes in cytokine profiles and lymphatic organ sizes. The results showed no significant differences in spleen, thymus, and lymph node weights, indicating a lack of overt systemic toxicity. Blood cytokine analysis revealed elevated levels of IL-6 and IL-1β in scaffolds with bacteria, suggesting a systemic inflammatory response, while TNF-α levels remained unaffected. Proteome profiling identified distinct cytokine patterns associated with bacterial colonization, including elevated inflammatory proteins and products, indicative of acute inflammation. Conversely, control scaffolds exhibited protein profiles suggestive of a rejection response, characterized by increased levels of cytokines involved in T and B cell activation. Our findings suggest that *Synechococcus* sp. PCC 7002 does not appear to cause significant systemic toxicity, supporting its potential use in biomedical applications. Further research is necessary to explore the long-term effects and clinical implications of these responses.

## 1. Introduction

Cyanobacteria, also referred to as blue-green algae, are pervasive in various aquatic environments, including freshwater, brackish, and marine ecosystems worldwide. These microorganisms play a crucial role as primary producers of oxygen, utilizing sunlight to convert carbon dioxide and water into oxygen and glucose photosynthetically [1]. In addition to oxygen, cyanobacteria synthesize a range of valuable substances, such as lipids, proteins, pigments, and bioactive compounds, which have garnered substantial international interest [2]. The products derived from these bacteria hold significant promise for sustainable biomass production for energy and pharmaceutical manufacturing. For instance, cyanobacteria are instrumental in the production of biofuels and biologically active compounds with antiviral, antibacterial, antifungal, and anticancer properties [3,4,5,6]. Certain strains are known to accumulate polyhydroxyalkanoates, offering a biodegradable alternative to conventional petrochemical-based plastics [7]. Furthermore, cyanobacterial hydrogen, recognized as a promising source of renewable energy, is now commercially available [8,9]. Beyond these applications, cyanobacteria are utilized in aquaculture, wastewater treatment, as food supplements, fertilizers, and for the production of secondary metabolites such as exopolysaccharides, vitamins, toxins, enzymes, and pharmaceuticals [10,11]. The unique characteristics and diverse applications of cyanobacteria position them as a pivotal resource, often heralded as the “green gold” of the future.

Cyanobacteria, despite their widespread use, are often associated with significant health risks. It is crucial to acknowledge that these microorganisms can produce a variety of toxic substances, which may pose dangers to human health. Cyanobacteria are capable of generating inherent toxins in quantities that can cause toxicity in mammals, including humans [12]. These cyanotoxins encompass both cyclic peptides and alkaloids. The cyclic peptide category includes toxins such as microcystins and nodularins, while the alkaloid category includes anatoxin-a, anatoxin-a(S), cylindrospermopsin, saxitoxins (STXs), aplysiatoxins, and lyngbyatoxin [13]. However, comprehensive data on the toxicity of cyanotoxins remain relatively limited. Additionally, the lipopolysaccharides (LPSs) present on the surface of cyanobacteria are also implicated in a range of adverse health effects in humans. LPSs are widely recognized as pathogenic components in many bacteria and are known to induce immune responses [14]. A specific strain known as *Synechococcus* sp. represents one of the primary groups of marine cyanobacteria and is regarded as a safe host for biotechnological applications because of its absence of toxin production and the presence of immunogenic LPS structures [15]. Consequently, this strain is well-suited for further biomedical research. Moreover, *Synechococcus* sp. PCC 7002, designed by Zhang et al. [16], offers several advantages over other microorganisms, including ease of manipulation, resistance to external factors, the ability to produce photosynthetic oxygen, and suitability for genetic modification.

Our research group is focused on enhancing the treatment of chronic wounds using these bacteria incorporated into collagen-based scaffolds [17,18,19]. The production of photosynthetic oxygen by these bacteria helps mitigate the hypoxic conditions of wound environments, while genetic modifications enable the release of growth factors directly into the wound site. A particular emphasis of our study is on the role of bacterially secreted hyaluronic acid in promoting lymphangiogenesis, with the goal of improving lymphatic drainage and reducing inflammation [17]. Therefore, cyanobacteria possess significant potential to promote the regeneration of chronic wounds.

Previous studies have successfully developed a scaffold bioactivated by cyanobacteria and conducted an in vitro investigation of its regenerative potential. Preliminary tests have demonstrated that the bacteria exhibit biocompatibility with human dermal cells [20]. The forthcoming phase of our study will focus on dermal applications, necessitating an evaluation of the initial effects of *Synechococcus* sp. PCC 7002 on mammalian hosts. It is, therefore, crucial to rigorously exclude any cytotoxic effects, rejection responses, and systemic immune reactions caused by the bacteria and the scaffolds employed. However, the current literature offers limited studies addressing the direct impact of *Synechococcus* sp. PCC 7002 on the immune system. Consequently, a comprehensive investigation into potential alterations in immune organs and cytokine levels in the blood is warranted. To this end, cyanobacteria were seeded into collagen-based scaffolds and implanted into bilateral full-thickness skin defects on the backs of mice. After 7 days, key immune organs (thymus, spleen, and lymph nodes) were explanted and quantitatively compared. Additionally, mouse blood was analyzed for changes in cytokine profiles and chemokine levels using ELISA and proteome profiling techniques.

## 2. Materials and Methods

### 2.1. Cell Culture of Cyanobacteria

Two types of cyanobacteria were used for the experiments. These included wild-type (SynWT) and transgenic *Synechococcus* sp. PCC 7002 cyanobacteria (strain SynHA12). Lifang Zhang and Tiago Toscano Selão generated the bacteria as part of their preliminary work and made it available to our working group [16]. The cyanobacteria were then kindly provided by the Biomedical Center within the Faculty of Biology at LMU University (Professor Nickelsen). Both types were cultivated on A-D7 medium agar plates supplemented with glucose (1 g/L) and chloramphenicol (10 μg/mL) at 30–50 μE·m^−2^·s^−1^ and 25 °C–30 °C. Every three weeks, the plates were refreshed. Strains SynHA12 and SynWT were used for all experiments since their genetic antibiotic resistance facilitated sterile culture conditions. Prior to each experiment, a liquid preculture was initiated by inoculating agar-grown cyanobacteria into 50 mL of A-D7 medium. It was supplemented with 1 g/L glucose, followed by incubation for three days under standard culture conditions (30 °C, 150 rpm, 30–50 μE·m^−2^·s^−1^). The transgenic strain overexpresses Pasteurella multocida hyaluronic acid synthase, which permits the production and secretion of hyaluronic acid. To induce hyaluronic acid synthesis, 1 mM IPTG (Isopropyl beta-D-thiogalactoside, Merck, Darmstadt, Germany) was introduced to the mutated cyanobacterial cultures, which were then resuspended in fresh medium to an optical density (OD750) of 1, as measured using an IMPLEN P300 Nanophotometer (Munich, Germany), and cultured for a minimum of seven days in the presence of IPTG. The wild-type (WT) bacteria were cultured under identical conditions. Detailed descriptions of the culture protocols were provided previously [17]. The cyanobacterial cell count was determined for all cell culture experiments using light microscopy (Primovert, Zeiss, Oberkochen, Germany) in conjunction with a Neubauer cell chamber. Subsequently, the cyanobacteria were cultured under standard mammalian cell culture conditions (37 °C, 5% CO_2_) with constant illumination by positioning them beneath a light source. To support photosynthetic growth, the used light emitted a complete spectrum of white light at a distance of 25 cm above the samples (32.25 μE·m^−2^·s^−1^, LED, Sebson, Dortmund, Germany).

### 2.2. Seeding of Cyanobacteria in DRM (Dermal Replacement Material)

The Integra bilayer matrix wound dressing (IDRT, Integra©Matrix Life Science Cooperation, Plainsboro, NJ, USA), composed of cross-linked collagen and glycosaminoglycans covered by silicone layers, was utilized as a dermal scaffold. This scaffold consists of a three-dimensional matrix that functions as an extracellular matrix, facilitating cellular proliferation and promoting collagen synthesis while gradually biodegrading and being replaced by autologous dermal tissue. Bacterial seeding was performed as previously described [17]. In summary, for all experiments, scaffolds were excised using a Ø12 mm biopsy punch (Pico Punch^®^ P1225, Acuderm^®^ Inc., Ft. Lauderdale, FL, USA), air-dried for 20 min on sterile gauze, and positioned with the collagen layer facing upwards in 6-well plates. A double-layered scaffold was employed, where collagen was overlaid with a silicone layer that acts as a temporary epidermis. Unless otherwise specified, cyanobacteria were seeded at a final density of 1 × 10^7^ cells per scaffold. For the bacterial seeding procedure, bacteria were transferred into a 50 mL tube and centrifuged at 4000× *g* for 5 min. The supernatant was subsequently discarded, and the bacteria were washed with 50 mL of PBS before being centrifuged at 4000× *g* for an additional 5 min. The resultant pellet was then resuspended in 50 μL of medium. Following this, 50 μL of Fibrin TISSEEL (Baxter GmbH, Unterschleißheim, Germany) was added in a 1:1 ratio, and the mixture was pipetted onto the scaffold. Finally, to ensure the fixation of the seeded bacteria within the scaffold, 50 μL of thrombin solution (TISSEEL, Baxter GmbH, Unterschleißheim, Germany) was applied to seal the bacteria within the matrix. The scaffolds were air-dried for one hour and then cultured in A-D7 medium, completely covering the scaffold at 30 °C and 5% CO_2_ under constant illumination for the desired duration. Scaffolds were prepared using genetically mutated bacteria with the addition of the promoter IPTG (Isopropyl beta-D-thiogalactoside) as well as non-mutated wild-type bacteria (WT), as previously described. As a negative in vivo control group (KO), empty scaffolds containing A-D7 medium without the integration of Syn7002 were prepared. In our earlier studies, we successfully demonstrated that the bacteria maintained sustained vitality and proliferation within the scaffold over a seven-day period, despite being fixed with fibrin. Furthermore, we confirmed the production and secretion of hyaluronic acid [17]. Additionally, we were able to demonstrate both in vitro and in vivo the microbial gene expression in bioactivated scaffolds colonized by cyanobacteria [21].

### 2.3. Stereoscopy

After the scaffolds were implanted, images were taken after 7 days of incubation on the back of mice using stereoscopes (Primovert, Zeiss, Oberkochen, Germany).

### 2.4. Full-Skin Defect Model

**Figure 1:**
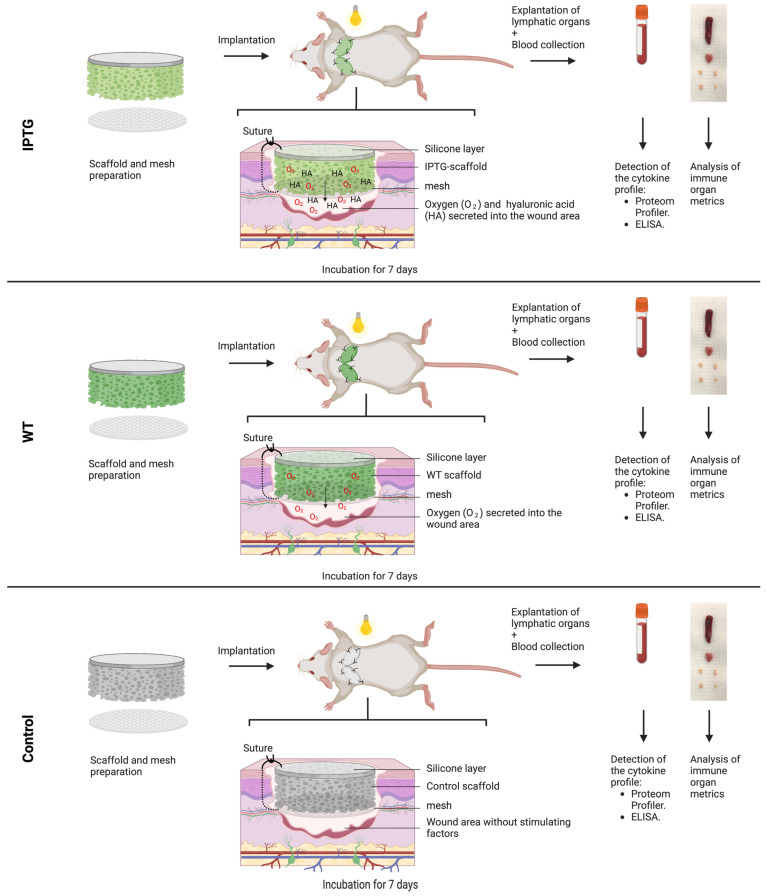
Schematic illustration of the experimental protocol. Scaffolds with a diameter of 12 mm were inoculated with either IPTG-induced bacteria (IPTG) or wild-type bacteria (WT). A control group (KO) consisted of scaffolds devoid of bacteria. These prepared scaffolds were subsequently implanted alongside a mesh into bilateral full-thickness defects on the dorsal side of mice for a duration of seven days. Post-experimentation, the animals were euthanized, and blood samples were collected for the analysis of key inflammatory markers. Additionally, the lymphatic organs were excised and compared for variations in their dimensions.

For the in vivo experiments, scaffolds were prepared as previously described. Prior to implantation, three scaffold groups were established as follows: IPTG, WT, and control scaffolds (KO). The scaffolds were incubated in an A-D7 medium for three days under the previously specified conditions. The experimental procedures outlined in this study received approval from the District Government of Upper Bavaria (ROB-55.2-2 532.Vet_02–19-96) and were conducted in accordance with the current German Animal Welfare Act (TierSchG). The experiments were conducted on female nude mice aged 6 to 8 weeks. The animals were euthymic and immunocompetent, with body weights ranging from 20 to 25 g (SHK1, Charles River, Sulzfeld, Germany). We created a bilateral full-skin defect measuring 10 mm under inhalation anesthesia (Isoflurane, Baxter Germany, Unterschleißheim, Germany). The implantation of scaffolds in mice was described in detail in previous publications [19]. The control, IPTG, and WT scaffolds were implanted and covered with a transparent dressing (V.A.C. Drape, KCI Medical Products, Wimborne Dorset, UK). To enhance photosynthesis in the scaffolds during daylight hours and stimulate oxygen production, the cages were fitted with a flexible LED module, providing a full spectrum of white light around the cages (32.25 μE·m^−2^·s^−1^, LED, Sebson, Dortmund, Germany). The mice were housed in the Central Animal Facility at the Medical Faculty of Ludwig-Maximilian-University Munich under a 12 h light/dark cycle, maintaining a constant temperature in individually ventilated cages. Consequently, the photosynthetic implants were subjected to no more than 12 h of light stimulation per day. The control animals were kept under identical light stimulation conditions. Seven days post-implantation, the mice were euthanized via cervical vertebrae dislocation. Whole blood was collected directly from the heart and allowed to clot undisturbed on ice for one hour. The clot was subsequently removed by centrifugation at 2000 g for 10 min at 4 °C. The serum was transferred to a clean test tube and stored at −80 °C for further analysis. A total of eight animals (16 scaffolds) per group were utilized in this study. Finally, the following lymphatic organs were excised from each mouse: the spleen, thymus, and one axillary and one inguinal lymph node from each side. The experimental setup is summarised in Figure 1.

### 2.5. Proteome Profiler

The proteome profiler Mouse Cytokine Array Panel A from R&D systems was used according to the manufacturer’s instructions. First, we centrifugated the EDTA-treated mouse plasma at 3000 g for 30 min, followed by storage at −20 °C. Reagent preparation involved bringing all components to room temperature and dissolving detection antibodies in 100 µL of distilled water. A 25× dilution of Wash Buffer was prepared by mixing 40 mL with 960 mL of distilled water, and Chemi Reagent 1 and 2 were combined 15 min before use and shielded from light. On Day 1, Array Buffer 6 was prepared for a 4-chamber plate, with nitrocellulose membranes placed in each chamber and incubated for 60 min on a plate shaker. Sample preparation included mixing 100 µL of EDTA-plasma with 1.4 mL of Array Buffer 4 in Eppendorf tubes. Each sample was then mixed with Mouse Cytokine Array Panel A detection antibody cocktail (15 µL initially, 10 µL post-stripping) and incubated for 60 min at room temperature. The incubation phase involved transferring prepared samples into chambers, closing them, and incubating them overnight at 4 °C. On Day 2, the membranes were washed thrice with 20 mL of 1× Wash Buffer for 10 min each on a plate shaker. Streptavidin-HRP was diluted 1:2000 in Array Buffer 6 and added to each chamber, where the membranes were incubated for 30 min at room temperature. Following a final wash with 1× Wash Buffer, the membranes were dried and prepared for chemiluminescence detection. Chemi Reagents 1 and 2 were mixed and applied to each membrane and incubated for 1 min; the excess reagent was absorbed, and the membranes were sealed for visualization using a chemiluminescence imager. Then, data analysis was performed using appropriate software. For membrane reuse, the stripping protocol involved washing membranes with Stripping Buffer thrice for 10 min each, followed by a 5 min wash with TBS-T. The membranes were dried and stored for subsequent use. Adjustments post-stripping included reducing the Detection Antibody Cocktail to 10 µL and Chemi Reagent volume to 500 µL.

### 2.6. ELISA

To detect the inflammatory cytokines IL-6, IL-1β, and TNF-α in mouse blood, we employed the ELISA Max™ Standard Sets from BioLegend according to the manufacturer’s instructions. For specimen handling, cell culture supernatants were centrifuged to remove debris, serum was collected using a serum separator tube with clotting and subsequent centrifugation, and plasma was collected using appropriate anticoagulant tubes followed by centrifugation. All samples were stored at temperatures below −20 °C to maintain stability, with strict avoidance of freeze/thaw cycles. For the assay procedure, the Capture Antibody was diluted 1:200 in Coating Buffer and added to a 96-well plate, which was then incubated overnight at 2 °C to 8 °C. Following wash steps with Wash Buffer, non-specific binding sites were blocked with Assay Diluent, and diluted standards (prepared from a lyophilized stock with Assay Diluent) along with samples were added to the plate and incubated at room temperature with shaking. After additional washes, a Biotinylated Detection Antibody and Avidin-HRP were sequentially added, each followed by incubation periods with shaking. The plate was then washed again, and substrate solution (TMB) was added and incubated in darkness until color development, which was subsequently stopped with Stop Solution. Absorbance was measured at 450 nm with optional correction at 570 nm. Storage conditions for kit components were strictly maintained between 2 °C and 8 °C. The results were calculated by plotting a standard curve using absorbance values of standards and interpolating cytokine concentrations of unknown samples using appropriate software for curve fitting.

### 2.7. Statistical Analysis

We performed all assays in at least three independent experiments, with at least two technical replicates for each experimental group. All data are presented as the mean with standard deviation. To compare differences between two distinct groups, we utilized Student’s *t*-test. Differences were considered significant if *p* ≤ 0.05 (ns: not significant; * *p* ≤ 0.05; ** *p* ≤ 0.01; *** *p* ≤ 0.001). All data were assessed for normal distribution before performing the *t*-test. Schematic representations were generated using the platform BioRender.

## 3. Results

### 3.1. No Systemic Effects on Lymphatic Organs after Bioactivated Scaffold Implantation

**Figure 2:**
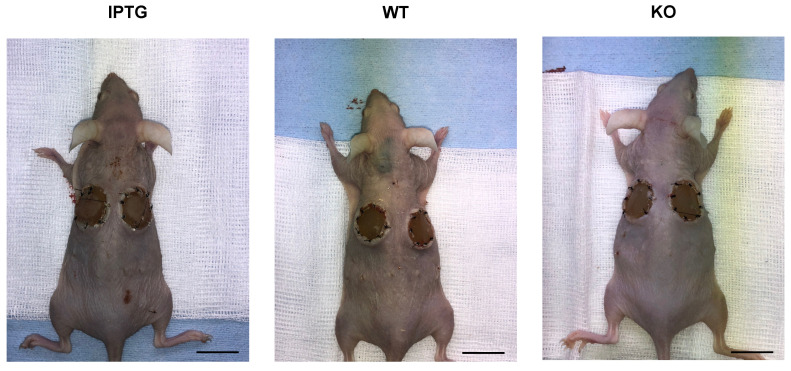
Representative images of the mice of all 3 groups (IPGT, WT, and KO) using a stereoscope directly after euthanization by cervical vertebrae dislocation 7 days after scaffold implantation. The area of the implantation site is locally non-irritant without typical signs of inflammation such as redness, swelling, or hyperthermia. The scale bar represents 2 cm.

**Figure 3:**
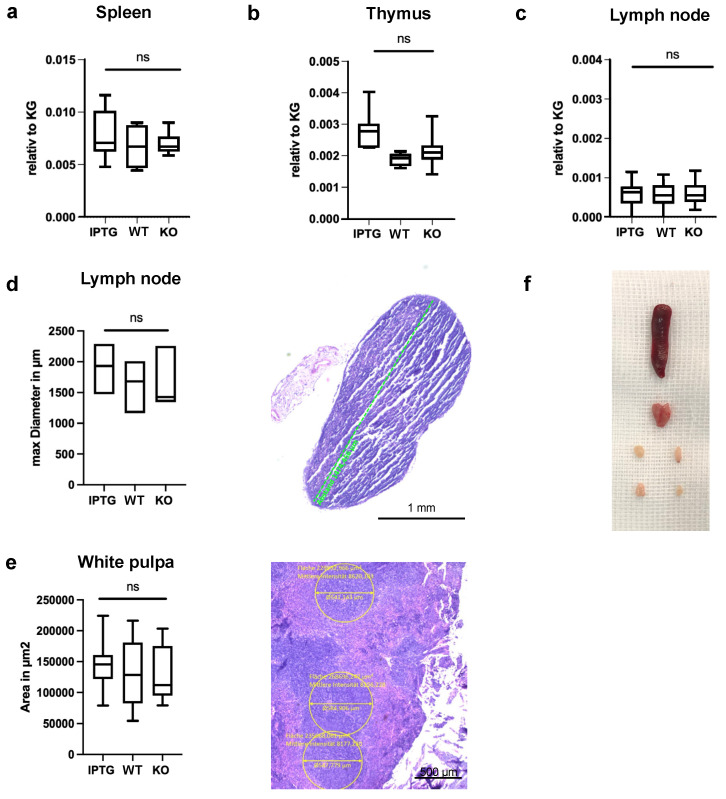
Analysis of immune organ metrics across different conditions. Demonstration of no systematic immune response 7 days after implantation of a scaffold colonized with IPTG-stimulated bacteria (IPTG), a scaffold with wild-type bacteria (WT), and a control scaffold (KO) in the absence of bacteria. We analyzed weight differences of the (**a**) spleen, (**b**) thymus, and (**c**) lymph nodes relative to the total weight of the mouse. (**d**) Left: We detected no significant differences in the maximal diameter of the axillar and inguinal lymph nodes. Right: Representative image of an inguinal lymph node in H&E staining. The scale bar represents 1 mm. (**e**) Left: The measurements of area in μm^2^ of the white pulp of the spleen revealed no marked differences in all groups. Right: Representative image of the white pulp in H&E staining. Magnification 5×. The scale bar represents 500 μm. Control scaffolds n = 10, colonized scaffolds n = 11, ns = not significant. (**f**) Representative image of the immune organs directly after explanation.

To evaluate the potential immune response to scaffolds colonized with bacteria, scaffolds were implanted in three distinct groups on the dorsal side of mice, as previously described. The first group contained bacteria induced to synthesize hyaluronic acid using the IPTG promoter, the second group consisted of non-induced wild-type bacteria, and the third group served as the control, containing scaffolds without any bacterial addition. Seven days post-implantation, there were no observable local signs of infection, such as erythema, edema, or hyperthermia (Figure 2). Throughout this study, the animals remained cardiopulmonary stable and exhibited no symptoms indicative of sepsis. Prior to analyzing inflammatory markers in mouse blood, we evaluated the systemic inflammatory response by examining the lymphatic organs. Thus, we excised critical immune system organs from the mice to infer potential systemic inflammation induced by the implanted bioactivated scaffolds, based on changes in their weight and size. Initially, we measured the weights of the spleen and thymus, as well as the axillary and inguinal lymph nodes. The mice used for the experiments all had a standard weight of 20–25 g. A gross anatomical comparison of the spleen, thymus, and lymph nodes is provided for visual reference (Figure 3f). First, we evaluated the relative weight of the spleen, normalized to body weight (KG), under three conditions as follows: IPTG, wild-type (WT), and control (KO) (Figure 3a). The results indicated no significant differences (ns) in relative spleen weight among the IPTG (Median: 0.0072, Q3: 0.010, Q1: 0.006), WT (Median: 0.0068, Q3: 0.008, Q1: 0.005), and control groups (Median: 0.007, Q3: 0.0074, Q1: 0.0068). Next, we examined the weight of the explanted thymus (Figure 3b). Similarly, no significant differences (ns) were observed in relative thymus weight across the IPTG (Median: 0.0028, Q3: 0.003, Q1: 0.0023), WT (Median: 0.0019, Q3: 0.002, Q1: 0.0017), and control groups (Median: 0.0021, Q3: 0.0022, Q1: 0.0019). Subsequently, we assessed the weight of the explanted lymph nodes, finding no significant differences among the IPTG (Median: 0.00075, Q3: 0.00085, Q1: 0.0004), WT (Median: 0.0007, Q3: 0.00085, Q1: 0.0004), and control groups (Median: 0.00071, Q3: 0.00085, Q1: 0.0005). We also measured the maximum diameter of the lymph nodes in micrometers under the IPTG (Median: 1953, Q3: 2278, Q1: 1565), WT (Median: 1671, Q3: 1997, Q1: 1202), and control conditions (Median: 1482, Q3: 2197, Q1: 1401) (Figure 3d). No significant differences (ns) in the maximum diameter of the lymph nodes were observed among the conditions. A representative histological section of a lymph node, stained to highlight structural features, is shown, with the green line indicating the maximum diameter. Finally, we examined the area of the white pulp in square micrometers (µm^2^) under the IPTG (Median: 149,501, Q3: 158,223, Q1: 124,993), WT (Median: 131,076, Q3: 176,145, Q1: 87,650), and control conditions (Median: 114,532, Q3: 170,342, Q1: 98,117) (Figure 3e). Again, no significant differences (ns) in the area of the white pulp were detected among the three groups. The histological section of the spleen highlights the white pulp area with annotated measurements. Across the various conditions (IPTG, WT, KO), no significant differences were observed in the relative weights of the spleen, thymus, and lymph nodes, as well as in the maximum diameter of the lymph nodes and the area of the white pulp. These findings indicate that scaffold implantation does not affect the size or weight of these immune organs.

### 3.2. Increased IL-6 and IL-1 Beta Profile after Bioactivated Scaffold Implantation

**Figure 4:**
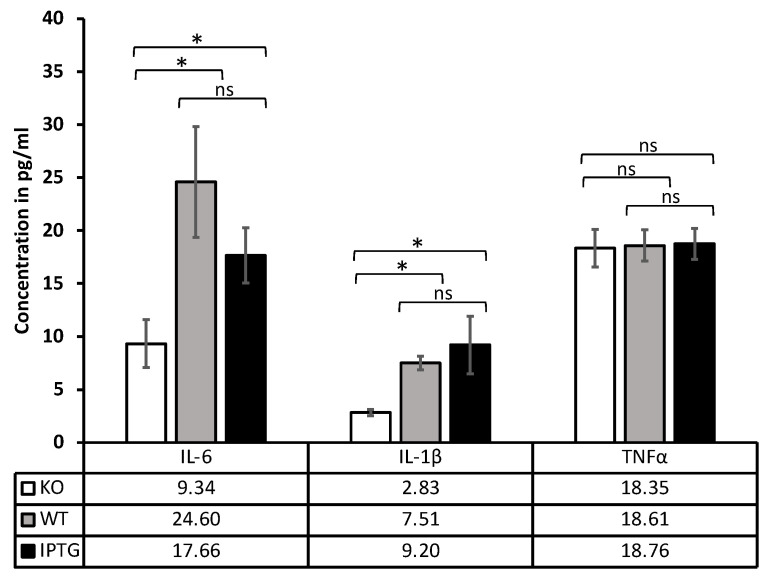
Detection of a systematic immune response 7 days after implantation of a scaffold colonized with IPTG-stimulated bacteria (IPTG), a scaffold with wild-type bacteria (WT), and a control scaffold (KO) in the absence of bacteria. Measurement of cytokine concentration in pg/mL by ELISA in mouse blood 7 days after scaffold implantation. The inflammatory cytokines detected are IL-6, IL-1β, and TNF-α. Cut off: IL-6 < 7.8 pg/mL, IL-1β < 6 pg/mL, TNF-α < 25 pg/mL, N ≥ 3; ns = not significant; * *p* < 0.5.

Seven days post-implantation of the scaffold into a full-thickness skin defect in mice, blood was collected as previously described and analyzed using ELISA to quantify the following key inflammatory markers: IL-6, IL-1β, and TNF-α (Figure 4). The IL-6 levels showed a marked increase in cytokine concentration in pg per ml in scaffolds colonized with bacteria compared with bacteria-free scaffolds (9.34 ± 3.1). Notably, a pronounced peak was observed in the wild-type bacteria group (24.60 ± 5.2), contrasted with the IPTG-stimulated bacteria group (17.66 ± 2.4). Conversely, IL-1β levels were generally lower, yet a distinct difference between colonized and non-colonized scaffolds persisted. Scaffolds with IPTG-stimulated bacteria exhibited concentrations of 9.20 ± 2.6, while those with wild-type bacteria showed 7.51 ± 0.45, with no statistically significant difference between these two groups. However, the control group demonstrated a significantly lower IL-1β concentration (2.83 ± 0.14). These findings suggest that cyanobacteria may elicit a systemic immune response. In contrast, TNF-α concentrations did not significantly differ among the three groups (IPTG 18.76 ± 1.7; WT 18.61 ± 1.9; KO 18.35 ± 2.0).

### 3.3. Shift in the Cytokine Profile after Bioactivated Scaffold Implantation

**Figure 5:**
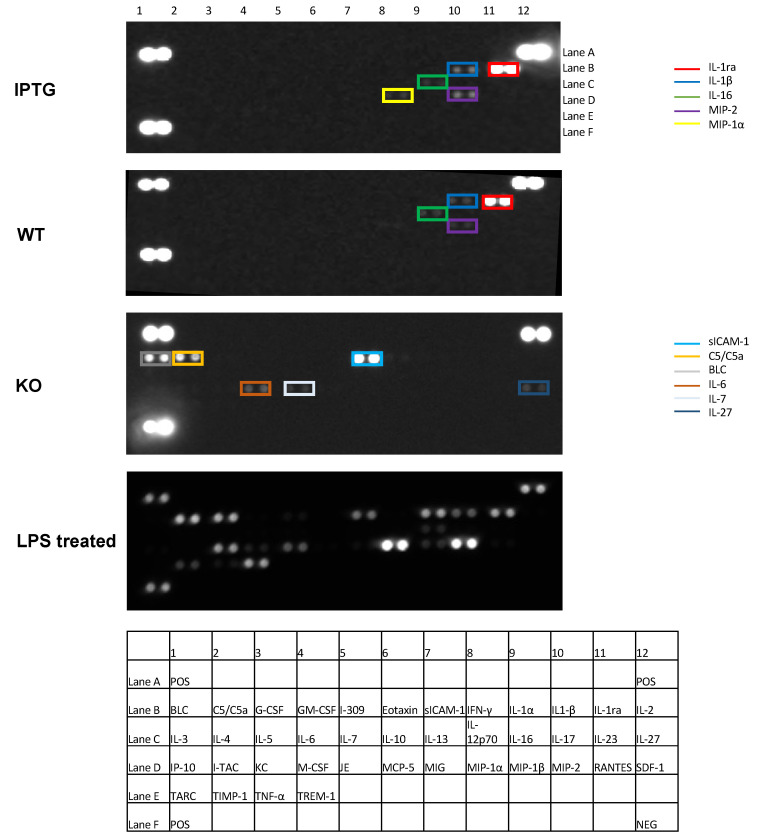
Detection of the cytokine profile 7 days after scaffold implantation in mouse blood using a proteome profiler. The following groups were analyzed: scaffolds with IPTG-stimulated bacteria (IPTG), scaffolds with wild-type bacteria (WT), control scaffolds (KO) in the absence of bacteria, and positive control (LPS treated) after LPS treatment. Significant differences exist in the cytokine profile between the scaffolds colonized with bacteria and the control scaffolds without bacteria. A table with the corresponding localization of the measured cytokines is attached. The positive control is shown in the top right, top left, and bottom left corners. The negative control is located at the bottom right. N > 3.

In our subsequent analysis, we examined the cytokine profile in the blood of mice 7 days post-scaffold implantation utilizing a proteome profiler. The focus was directed towards the most recognized and relevant markers of inflammation, which are delineated in the table presented in Figure 5. This figure also illustrates the specific localization of each cytokine. The cytokine profiles of all three experimental groups were compared against a positive control treated with LPS. The brightness of each cytokine spot correlates with its measured concentration. A detailed examination reveals that the cytokine profiles of the two groups with bacteria-colonized scaffolds are similar, while the control group without bacteria exhibits significant differences. Notably, the inflammatory markers IL-1ra, IL-1β, IL-16, and MIP-2 are detectable in the blood of mice implanted with bacteria-colonized scaffolds. The luminescence, and hence, the measured concentration of these cytokines, is significantly higher in the group stimulated with IPTG. Additionally, MIP-1α is detected in the IPTG-stimulated group but is absent in the group with wild-type bacteria. In contrast, the cytokine profile of the control group, devoid of bacterial colonization, is markedly different. The aforementioned cytokines are undetectable, and a distinct set of inflammatory markers, namely, sICAM-1, C5/C5a, and BLC, exhibit increased luminescence. Meanwhile, IL-6, IL-7, and IL-27 are observed with reduced luminescence intensity. These findings suggest that scaffolds colonized with cyanobacteria induce a distinct inflammatory response in the mouse organism 7 days post-implantation, compared with collagen-based scaffolds without bacterial colonization. This difference manifests as a significant shift in the cytokine profile.

## 4. Discussion

Cyanobacteria, also known as “blue-green algae,” are widespread bacteria found in freshwater systems worldwide [22]. Recently, there has been a growing societal interest in natural ingredients and nature-based products, such as creams and peelings [23]. Algae and bacteria have attracted significant attention for their potential in tissue regeneration in medical applications. Cyanobacteria are particularly notable because of their ability to produce oxygen through photosynthesis and their ease of genetic modification to synthesize growth factors essential for skin regeneration. Unlike the microalga Chlamydomonas reinhardtii, which was previously used by Schenck et al. [19], unicellular, euryhaline, and mixotrophic cyanobacteria offer numerous benefits for skin applications, particularly in managing inflammatory tissue and facilitating experimental manipulations. A key feature of *Synechococcus* sp. strain PCC 7002 is its rapid proliferation, with an average doubling time of 4 h, which is twice as fast as many other microorganisms [24]. This characteristic suggests that incorporating *Synechococcus* sp. strain PCC 7002 could greatly enhance the efficiency and effectiveness of skin regeneration processes, making it a promising candidate for future biomedical applications, especially in developing advanced therapeutic products for skin repair and regeneration. Given their anticipated compatibility with mammalian systems, algae are of particular interest, prompting further research into the effects of cyanobacteria on mammalian hosts. Cyanobacteria are scientifically significant because of their diverse applications; however, their interactions with mammalian immune systems remain underexplored. Our study focuses on assessing the safety of cyanobacteria application in a murine model, specifically examining its effects on systemic inflammatory responses.

Cyanobacterial lipopolysaccharides (LPSs) are frequently documented in the scientific literature as potential toxins that may induce a spectrum of adverse health effects in humans, encompassing dermatological manifestations, gastrointestinal disturbances, respiratory complications, allergic responses, headaches, and febrile conditions [14,25,26]. The pathophysiology of cyanobacteria-related illnesses is typically classified under intoxication rather than infection, a classification supported by the acute onset of clinical symptoms following exposure and the absence of an incubation period or subsequent secondary infections [27]. The existing evidence does not substantiate the hypothesis that cyanobacterial LPS elicits dermatological reactions in individuals with otherwise normal health status [28,29,30]. In the literature, few instances describe cyanobacteria as invasive or infectious entities. Rank et al. hypothesized a low-virulence, chronic infestation by heterotrophically growing cyanobacteria as a potential etiological factor for arteriosclerosis in humans and homeothermic animals, a theory based on ecological data and analyses of disease distribution patterns [31,32,33]. However, this hypothesis has not gained traction in subsequent scientific discourse. Cyanobacteria possess a modified form of lipopolysaccharides (LPSs) that is structurally distinct from the conventional LPSs found in other Gram-negative bacteria and is characterized by reduced toxicity [34]. *Synechococcus* species represent a significant group within marine cyanobacteria [16]. Specifically, the LPS of *Synechococcus* strains lacks the typical heptose and keto-deoxyoctulosonate (KDO) sugars in the inner core region. Moreover, the lipid A component of their LPS is devoid of phosphates, contains a single galacturonic acid residue, and is composed of odd-chain hydroxylated fatty acids [34]. Although *Synechococcus* sp. PCC 7002 is also capable of producing LPSs, the immune response elicited by LPSs from various *Synechococcus* species is either negligible [35] or at least three orders of magnitude lower than that provoked by LPSs from other Gram-negative bacteria [14]. As a result, *Synechococcus* species are considered safe hosts for biotechnological applications [36].

In this study, we evaluated the systemic inflammatory response following the implantation of scaffolds colonized with cyanobacteria into full-thickness skin defects in mice. Three distinct experimental groups were analyzed as follows: bacteria engineered to synthesize and secrete hyaluronic acid upon induction with the IPTG promoter, wild-type bacteria lacking this synthetic capability, and control scaffolds devoid of bacteria. The observation period lasted for seven days. The study period coincided with the study by wang et al., which demonstrated a significant increase in the CD8 T cell concentration after just 7 days of a bacterial infection with listeria monocytogenes [37]. Post-explantation, inflammation was assessed by analyzing changes in mouse blood cytokine profiles and alterations in primary lymphatic organ dimensions, including the spleen, thymus, and axillary and inguinal lymph nodes. Among all physiological systems, the immune system exhibits the highest likelihood of exposure and susceptibility to toxic materials. Consequently, toxins affecting other organs or systems are expected to also impact the immune system, potentially causing exacerbated adverse effects. Because of the paucity of in vivo experimental evidence concerning cyanobacteria, we initiated this study. The primary objective was to elucidate the extent to which cyanobacteria, the hyaluronic acid they produce, and the scaffold itself induce systemic inflammation in a mammalian host. Our study is the first to report the effect on the mammalian immune system in vivo by photosynthetically genetically modified cyanobacteria.

Initially, we examined the lymphatic organs seven days post-implantation of the scaffolds. For this analysis, the aforementioned organs were explanted and documented photographically (Figure 2). The primary objective was to evaluate morphological differences, particularly in terms of organ weight in grams. No significant differences were observed in the weights of the spleen, thymus, or lymph nodes among the three groups (Figure 3a–c). This finding suggests that the implantation of bacteria-colonized scaffolds does not elicit any detectable systemic inflammatory response. The lack of weight differences in the spleen, thymus, and lymph nodes among the groups indicates an absence of clinical toxicity. Regular veterinary assessments were conducted, employing a standardized scoring system. No abnormal vital parameters or signs of sepsis were detected in any of the animals. Contrastingly, Shen et al. reported a marked alteration in lymphatic organs following the administration of cyanotoxin from cyanobacteria blooms in mice [38]. Depending on the treatment dose, it led to an increase in spleen ratios in mice, while the thymus remained unaffected. The livers showed swelling and intrahepatic hemorrhages. At this point, it must be mentioned that a different strain of bacteria was used in the above-mentioned study. Further, an effect on the systemic immune system was only seen at higher doses. In addition to organ weight, we measured the diameters of the axillary and inguinal lymph nodes. Similarly, no significant differences were observed, further supporting our hypothesis that there was no systemic reaction (Figure 3d). To enhance the validity of our findings, we analyzed the white pulp area of the spleen. In mice, rats, and humans, the spleen comprises white pulp embedded within red pulp. In the white pulp, T and B lymphocytes aggregate to form periarteriolar lymphatic sheaths and follicles surrounding the central arteries. The red pulp consists of reticular connective tissue housing various blood cells. Our analysis revealed no significant differences in the diameter of the white pulp across the three groups. While direct comparison with the human spleen is limited, it is noteworthy that the spleens of mice and rats possess a distinct B cell compartment, the marginal zone, situated between the white and red pulp, an area absent in the human spleen [39]. In an oral toxicology investigation, mice were subjected to a gavage administration of *Synechococcus* sp. PCC 7002 at a dosage of 10 g per kilogram of body weight [15]. The study revealed the absence of any treatment-related mortality or clinical manifestations, which is consistent with our findings. Additionally, no detrimental effects were observed concerning food intake, organ coefficients, or histopathological alterations in the liver, kidneys, or small intestine [15]. Taken together, seven days post-implantation of bacteria-colonized scaffolds, no significant morphological or weight differences in lymphatic organs were observed in this study, indicating an absence of systemic inflammatory response or clinical toxicity.

Next, we investigated the immune response 7 days post-scaffold implantation by analyzing blood samples from mice. Upon the entry of bacteria into the bloodstream, a rapid inflammatory response is typically activated. Innate immune cells identify evolutionarily conserved structures on bacteria, known as pathogen-associated molecular patterns (PAMPs), as well as endogenous stress signals, referred to as damage-associated molecular patterns (DAMPs), through germline-encoded pattern recognition receptors (PRRs). These receptors are predominantly expressed by macrophages and dendritic cells; however, they are also present on a variety of immune and non-immune cells, including neutrophils, lymphocytes, fibroblasts, and epithelial cells. The activation of PRRs triggers proinflammatory and antimicrobial responses by stimulating the release of various cytokines, with the production of acute phase proteins (APPs) serving as a critical component of the inflammatory response [40]. The primary proinflammatory cytokines, TNF-α, IL-1β, and IL-6, are swiftly released from monocytes and macrophages upon PRR activation [41]. TNF-α and IL-1β facilitate the expression of leukocyte adhesion molecules on the vascular endothelium, promoting the rapid recruitment of leukocytes to inflammation sites [42,43,44]. TNF-α, by binding as a trimer to either the 55 kDa TNFR-1 or 75 kDa TNFR-2 cell membrane receptors, contributes to local vasodilation (rubor, calor), vascular leakage (resulting in swelling), and necrosis or apoptosis via various signaling pathways [45,46,47]. IL-1β induces inflammatory hyperalgesia and the production of IL-6 [48,49,50]. IL-6, a 26 kD secreted protein, is crucial for triggering the production of acute phase proteins by hepatocytes, thus playing a significant role in host defense by stimulating hematopoiesis and immune responses [51,52,53].

To analyze the cytokine levels, blood samples were collected as previously described, and the cytokine profile was assessed. Initially, we focused on the cytokine levels of IL-6, IL-1β, and TNF-α utilizing ELISA. Our study found significantly elevated IL-6 levels in scaffolds colonized with bacteria compared with those without. It is important to note that IL-6 levels were consistently low across all three groups. Although baseline IL-6 levels are generally expected to range between 5 and 20 pg/mL, these values can vary significantly depending on factors such as mouse strain, age, sex, and experimental conditions, rendering reliable reference values difficult to establish [54]. Following inflammation or stimulation, IL-6 levels typically exceed 50 pg/mL using the same kit [55,56]. In a study by Chen et al. [57], an average IL-6 concentration of 46.2 ± 8.9 pg/mL was measured in healthy mice (7 weeks old, male), suggesting that while the bacteria-colonized scaffolds lead to an increase in IL-6, they do not provoke a systemic inflammatory response. These results are consistent with the findings of Williams et al. [58], who observed a minor increase in cytokine levels 4 h after intravenous injection of *Synechococcus* elongatus in Wistar rats; however, there was no significant difference from the saline-injected control group. It is crucial to note that the cytokine profiles of mice and rats differ significantly, and direct comparisons are therefore not feasible [59]. In addition, the bacterial strain differs from the bacteria used in this study, which limits the comparability because of different properties. The reduced interleukin-6 (IL-6) concentration in the IPTG group compared with the WT group may be attributed to the anti-inflammatory properties of the hyaluronic acid produced by the bacteria [60]. This finding supports the potential efficacy of employing genetically modified cyanobacteria in wound healing applications. However, this effect was absent in the measurement of IL-1β. Our analysis of IL-1β revealed a stimulatory effect of the bacteria-laden scaffolds compared with the control scaffolds, suggesting a systemic response. However, it is essential to consider the reference values in this context. Other studies have reported IL-1β levels exceeding 10 pg/mL in healthy mice, indicating that the bacterial surface may not have been recognized as a pathogen, thereby failing to trigger systemic inflammation through IL-1β [61,62]. No significant differences were observed between genetically modified and wild-type bacteria, indicating that the secreted hyaluronic acid, despite its potential pro- and anti-inflammatory effects depending on its molecular length, does not induce any stronger additional systemic infection response in this mouse model [63]. In contrast, recent studies have explored the properties of microcystins, a class of hepatotoxins consistently synthesized by cyanobacteria species belonging to the genera *Microcystis*, *Anabaena*, *Nostoc*, and *Oscillatoria* [64]. In fact, microcystins exert significant effects on the immune system [65] by influencing the production of interleukin-1 (IL-1) and tumor necrosis factor-alpha (TNF-α) [66,67]. However, we also observed further differences from the previous studies. Our investigations included TNF-α, which revealed no significant differences among the three groups, supporting our hypothesis that cyanobacteria do not provoke inflammation, cell apoptosis, or tissue necrosis. TNF-α typically increases in the context of inflammation and is crucial for the migration and adhesion of neutrophils [68]. The measured TNF-α levels are substantially lower than the baseline level of approximately 43.7 pg/mL reported in other studies involving healthy mice, which counteracts a systemic infection [57,69]. In line with this, Williams et al. [58] reported no significant increased TNF-α levels 4 h following intravenous injection of *Synechococcus* elongatus in Wistar rats compared to saline injections. Taken together, the results indicated a possible low systemic inflammatory response, particularly elevated IL-6 and IL-1β levels, due to bacterial colonization on the scaffolds, while TNF-α levels showed no significant change. However, it has to be emphasized that the measured elevated cytokine levels were only marginally above detection thresholds with a moderate concentration, which contradicts a strong inflammatory reaction in mammalian hosts.

Following the observed elevation in cytokine concentrations of IL-6 and IL-1β via ELISA, our research focus extended to a comprehensive protein profile analysis in murine blood samples to elucidate the potential induction of systemic inflammation. Systemic inflammatory responses are characterized by the interaction of multiple signaling molecules. Therefore, we augmented our study with a proteome profiling approach. In addition to the previously mentioned cytokines, critical mediators of acute inflammatory responses include IL-11, IL-8, G-CSF, and GM-CSF [59,70]. However, chronic inflammation, often associated with significant tissue damage and a progressive alteration in cell type at the site of inflammation, can emerge as the disease progresses. The cytokines implicated in chronic inflammatory responses, as identified through proteome profiling, include IL-3, IL-6, TNFβ, IL-4, IL-5, IL-7, IL-9, IL-10, IL-13, and IFN [70]. Additionally, the activation of complement component C5/C5a and macrophage inflammatory proteins (MIPs) can be assessed using the proteome profiler. Our findings indicate a distinct cytokine profile between the bacteria-colonized scaffolds and control scaffolds devoid of bacterial colonization. The presence of bacteria appears to have activated a unique set of proteins. In the scaffolds colonized with bacteria (both IPGT and WT), cytokines primarily associated with acute phase inflammation were observed, suggesting a role in recruiting additional inflammatory cells via chemotaxis. Contrary to our expectations, the levels of G-CSF and GM-CSF did not exhibit an increase. However, an elevation in MIPs, which are secreted by macrophages and monocytes and contribute significantly to chemotaxis, was detected. A predominant increase in inflammatory protein concentration was observed in the control group without bacterial presence, possibly involved in a rejection response. The cytokines IL-7 and IL-27 detected in this context are primarily involved in the induction of B and T cell development, recruitment, activation, and differentiation. These findings may also explain the increase in B-lymphocyte chemoattractant (BLC). Similar results were reported by Swanson-Mungerson et al. [22] in their study of the cyanobacterium *Oscillatoria* sp., where cyanobacterial lipopolysaccharides (LPSs) induced B cell proliferation, upregulated MHC II and CD86 expression, and enhanced antigen uptake. Furthermore, an increased concentration of the complement activation product C5a was measured, which is known to facilitate the recruitment of phagocytic neutrophils and the activation of mast cells, leading to the release of proinflammatory mediators [71]. This supports the hypothesis of a possible rejection response. In our study, we detected an elevated level of soluble intercellular adhesion molecule-1 (sICAM-1) in the control group. sICAM-1 is a pivotal molecule involved in diapedesis, a process that facilitates the migration of leukocytes across the vascular endothelium and is integral to the inflammatory response [72]. Recent research has demonstrated that sICAM-1 can induce a two- to six-fold enhancement in the proliferative response of T cells [73]. This finding bolsters our hypothesis regarding the activation of T and B cells induced by the scaffold in the absence of bacterial involvement. In conclusion, the results revealed distinct cytokine profiles and systemic inflammatory responses between the bacteria-colonized and control scaffolds, with the former showing proteins that presumably might be associated with low acute inflammation and the latter indicating a potential rejection response, including the activation of T and B cells.

The limitations of this study include the small sample sizes and the brief observation period, which was restricted to a single measurement point after seven days in consideration of animal welfare. Consequently, it is imperative to conduct additional studies that investigate the long-term effects.

## 5. Conclusions

Our study suggests that *Synechococcus* sp. PCC 7002, when used in scaffolds for skin regeneration, may not induce significant systemic inflammation or clinical toxicity in a murine model. While elevated levels of IL-6 and IL-1β were observed, indicating a potential acute inflammatory response, severe adverse effects like necrosis or organ damage were not observed. *Synechococcus* sp. PCC 7002 did not appear to be strongly immunogenic or cause clinically significant disease during the investigated time period. Additionally, transgenic *Synechococcus*, which produces hyaluronic acid, did not seem to exhibit increased immunogenicity compared to the wild-type bacteria. Overall, these preliminary findings suggest potential safety for biomedical applications, but more research is needed to resolve the data on inflammation and fully understand the long-term effects and immune interactions.

## Data Availability

The original contributions presented in the study are included in the article, further inquiries can be directed to the corresponding author.
